# Molecular Characterization of MHC Class I Alpha 1 and 2 Domains in Asian Seabass (*Lates calcarifer*)

**DOI:** 10.3390/ijms231810688

**Published:** 2022-09-14

**Authors:** Zhixuan Loh, Xuelu Huan, Sunita Awate, Markus Schrittwieser, Laurent Renia, Ee Chee Ren

**Affiliations:** 1 Singapore Immunology Network, Agency for Science, Technology and Research (A*STAR), Singapore 138648, Singapore; 2 A*STAR Infectious Diseases Labs (A*STAR ID Labs), Agency for Science, Technology and Research (A*STAR), Singapore 138648, Singapore; 3 UVAXX Pte Ltd., Singapore 159546, Singapore; 4 Lee Kong Chian School of Medicine, Nanyang Technological University, Singapore 636921, Singapore; 5 School of Biological Sciences, Nanyang Technological University, Singapore 637551, Singapore; 6 Yong Loo Lin School of Medicine, National University of Singapore, Singapore 117597, Singapore

**Keywords:** Asian seabass, *Lates calcarifer*, MHC class I, sequence variability, polymorphism

## Abstract

The Asian seabass is of importance both as a farmed and wild animal. With the emergence of infectious diseases, there is a need to understand and characterize the immune system. In humans, the highly polymorphic MHC class I (MHC-I) molecules play an important role in antigen presentation for the adaptive immune system. In the present study, we characterized a single MHC-I gene in Asian seabass (*Lates calcarifer*) by amplifying and sequencing the MHC-I alpha 1 and alpha 2 domains, followed by multi-sequence alignment analyses. The results indicated that the Asian seabass MHC-I α1 and α2 domain sequences showed an overall similarity within Asian seabass and retained the majority of the conserved binding residues of human leukocyte antigen-A2 (HLA-A2). Phylogenetic tree analysis revealed that the sequences belonged to the U lineage. Mapping the conserved binding residue positions on human HLA-A2 and grass carp crystal structure showed a high degree of similarity. In conclusion, the availability of MHC-I α1 and α2 sequences enhances the quality of MHC class I genetic information in Asian seabass, providing new tools to analyze fish immune responses to pathogen infections, and will be applicable in the study of the phylogeny and the evolution of antigen-specific receptors.

## 1. Introduction

The Asian seabass (*Lates calcarifer,* Bloch 1790), also known as barramundi, is a commercially important fish species that is widely distributed in the Indo-Pacific Asia region, including Singapore and Northern Australia [1,2]. As the Asian seabass industry expands and production increases, commercial farmers have been battling emerging infectious diseases that affect the survival of farmed livestock [3,4,5,6,7,8]. Therefore, it is important to understand and characterize the immune system in order to combat this over-arching problem.

Classical major histocompatibility complex class I (MHC-I) molecules play pivotal roles in initiating an adaptive immune response against pathogens [9,10]. Structurally, the classical MHC-I molecules consist of three extracellular domains, where the α1 and α2 domains are involved in peptide binding, and the membrane α3 domain interacts with β2-microglobulin and provides molecular stability [11]. A major feature of these classical MHC-I molecules is the extensive alleles polymorphism predominantly found within the peptide binding α1 and α2 domains to help ensure the survival of the population by displaying a broad range of peptides to T cell receptors [12,13].

Currently, six MHC-I lineages, U, Z, S, L, P, and H, have been described in teleost fish [14,15,16,17,18,19]. These lineages are defined by polymorphisms, expression patterns, and peptide binding ability. Among the lineages, only the U lineage has been well studied at the molecular and functional levels due to its binding abilities [20]. While little is known about the nonclassical MHC-I lineages Z, S, L, P, and H, some studies have suggested that the Z lineage may bind to peptides with modification to its N-terminal [17,21].

In Asian seabass, predicted MHC-I sequences have been obtained from a single Asian seabass using next-generation sequencing [22]. However, the lineages of these sequences have not been validated, and the polymorphisms in MHC-I molecules have not been explored. Therefore, in the present study, we sequenced and characterized the peptide binding regions of a single MHC-I gene in Asian seabass obtained from Singapore and Australia.

## 2. Results

### 2.1. Amino Acid Sequences of Asian Seabass MHC-I α1 and α2 Domains

**Figure 1:**
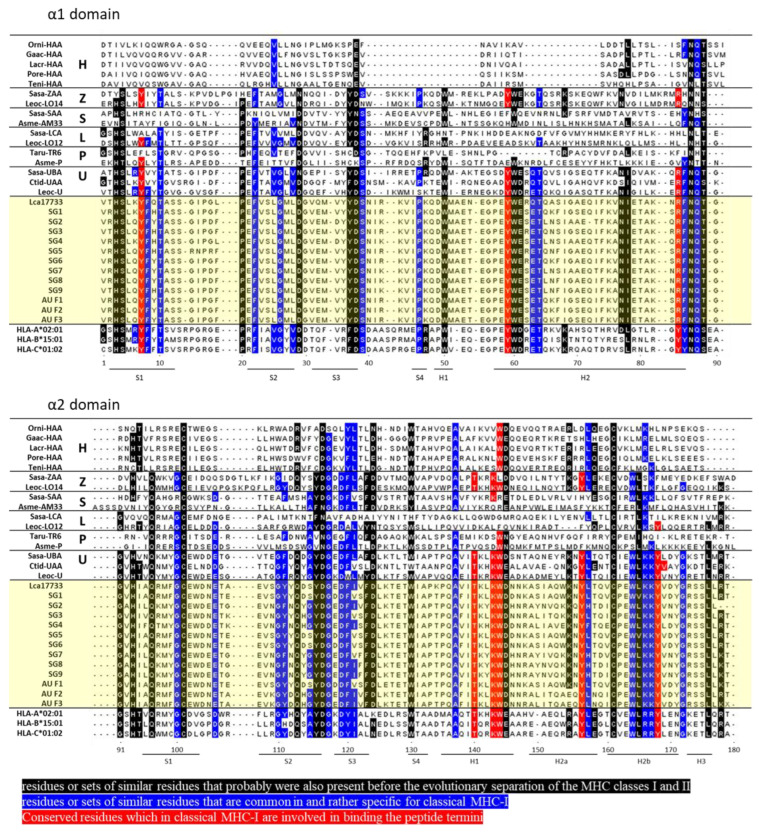
Alignment of Asian seabass MHC-I α1 and α2 amino acid sequences with other representative MHC-I sequences. Multiple sequence alignment of Asian amino acid sequences with other representative fish MHC-I and human HLA sequences. Black indicates similar residues that were probably found before the molecular evolution of MHC classes I and II, blue indicates similar residues that are common in classical MHC-I, and red indicates conserved binding residues in classical MHC-I. The sequences are divided into α1 and α2 regions, which correspond to exon 2 and exon 3, respectively. The residue positions indicated below the alignment are based on the HLA-A2 protein. Structural indications S denotes the β-strand and H denotes the helix based on the pHLA-A2 structure (PDB database accession 3PWN). The sequence references are as follows: Orni (Oreochromis niloticus, Nile tilapia) -HAA (TSA: GBAZ01123113); Gaac (Gasterosteus aculeatus, Stickleback) -HAA (DW655318); Lacr (Larimichthys crocea, Yellow croaker) –HAA (XP_010741942.1); Pore (Poecilia reticulata, Guppy) -HAA (XP_008432358.1 and TSA: GFHH01045885); Teni (Tetraodon nigroviridis, Tetraodon) -HAA (CAG07665.1); Sasa (Salmo salar, Atlantic salmon) -SAA (ACY30362.1), -ZAAa (ACX35596.1), and -UBA (*0301, XP_014032819); Leoc (Lepisosteus oculatus, Spotted gar) -LO14 (TSA: GFIM01040660), -LO12 (JH591577:52,184-56,541), and -U (TSA: GFIM01032149); Ctid (Ctenopharyngodon idella, Grass carp) -UAA (5H5Z-A); Asme (Astyanax mexicanus, Mexican tetra) -AM33 (ENSAMXG00000017444) and -P (TSA: GFIF01000014); Taru (Takifugu rubripes, Fugu) -TR16 (Scaffold_497:44,782-49,340); HLA (Human leukocyte antigen) -A2 (AAA76608.2); -B15:01 (HG794370.1), -C1:02 (HG794388.1), and Lca17733 (Lates calcarifer, Asian seabass) (XM_018684417). SG denotes samples obtained from Singapore and AU denotes samples obtained from Australia.

The classical MHC-I heavy chain gene is organized into eight exons with distinct functional domains [23,24]. To sequence the peptide-binding regions formed by the α1 and α2 domains coded by exon 2 and exon 3, respectively, RNA was isolated and cDNA was generated from the muscle tissues of Asian seabass. Exon 2 and exon 3 were amplified using PCR with primers designed on exon 1 and exon 4. The nucleotide sequences obtained from 12 Asian seabass (9 Asian seabass from Singapore and 3 Asian seabass from Australia) (Figure S1) were translated using the EMBOSS Transeq tool into protein sequences before aligning using Cluster Omega [25]. The amplified α1 domain (exon 2) comprises 87 amino acids and the α2 domain (exon 3) comprises 93 amino acids (Figure 1).

**Figure 2:**
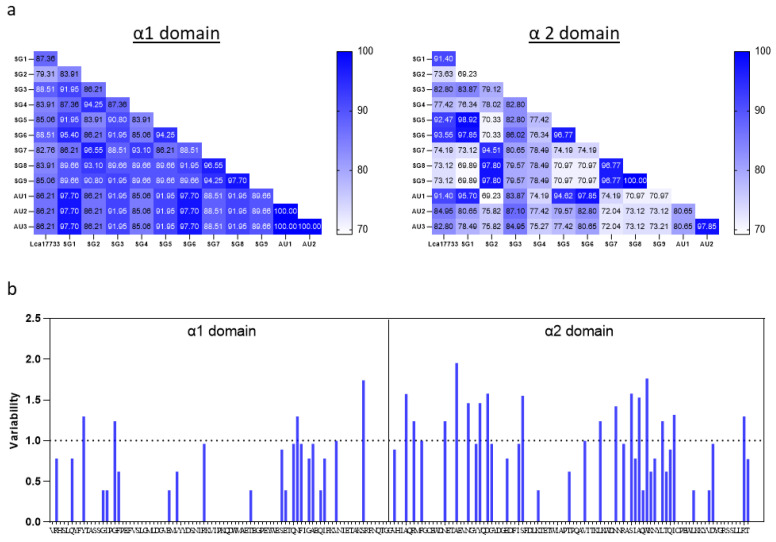
Amino acid identity and sequence variability within the Asian seabass α1 and α2 domains. (**a**) Heatmap of amino acid similarity values for Asian seabass and Lca17733 MHC-I α1 and α2 domains. (**b**) Sequence variability calculated using the variability metric (*V*) analysis based on the Shannon entropy equation. Sites with *V* >1 are considered as highly polymorphic in the sequence alignment of the Asian seabass and Lca17733 MHC-I α1 and α2 domains.

Comparing Lca17733 and the sequences from the 12 Asian seabass, our results indicated that the amino acid sequence variation among the α1 domain with pairwise comparisons ranged from 12.64 to 20.69%, with ±s.e.m. of 9.84 ± 0.522%, and amino acid sequence variation among the α2 domain with pair-wise comparisons ranged from 6.45 to 26.88%, with ±s.e.m. 19.17 ± 1.04%. This suggests that the sequences of the MHC-I α1 domain were more conserved as compared with those of the MHC-I α2 domain (Figure 2a). Furthermore, analysis using the variability metric (*V*), where *V* > 1 indicates highly polymorphic sites, identified 4 sites in the α1 domain and 16 sites in the α2 domain (Figure 2b). Genetic differentiation found between Asian seabass obtained from Australia and Singapore was not observed (Figure 2a).

The MHC-I α1 and α2 domain sequences were then compared with representative sequences of other MHC-I lineages found in teleost fishes and with the human MHC-I molecules HLA-A2, HLA-B15, and HLA-C1 (Figure 1). The highly conserved eight residues Y7, Y59, Y/R84, T143, K146, W147, Y159, and Y171, thought to be important to anchor the N- and C-terminal peptide ends [26,27,28], are highlighted in red (Figure 1). The Asian seabass MHC-I in all 12 fishes possessed all of the eight peptide binding termini residues, including residue R84, which is common in non-mammalian vertebrates such as sharks and teleost fish, instead of Y84, found in mammals and some reptiles [27,29] (Figure 1). Similar residues that were (1) probably found before the molecular evolution of MHC classes I and II (indicated in black) [30,31] or (2) common in classical MHC-I (indicated in blue) [19,30] were also found in the Asian seabass MHC-I α1 and α2 domain sequences, albeit with <11 substitutions out of the 70 highlighted conserved residues (Figure 1). Based on the length and pattern expression, the Asian seabass MHC-I showed an overall similarity to classical human MHC-I molecules and may have a similar structure.

### 2.2. Phylogenetic Analyses of Asian Seabass MHC-I α1 and α2 Domains

**Figure 3:**
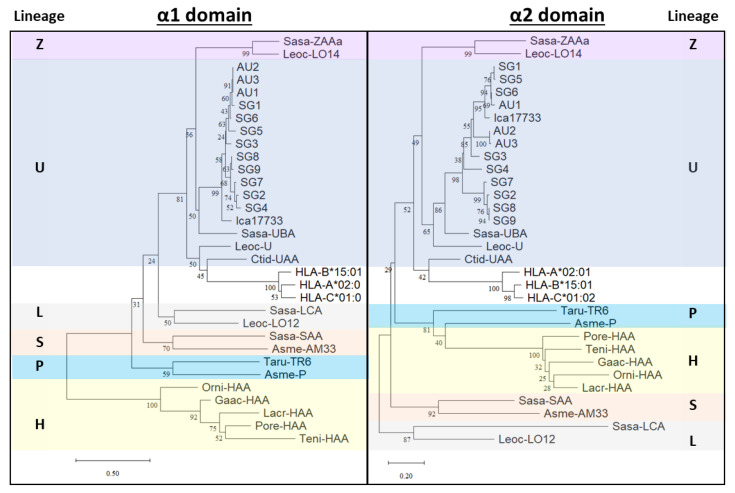
Phylogenetic trees based on α1 and α2 domain amino acid sequences of Asian seabass and other representative teleost fishes’ MHC-I molecules. Neighbor-joining tree [32] of α1 (left tree) and α2 (right tree) domain sequences. The trees are drawn to scale, with branch lengths indicating the number of amino acid substitutions per site. The percentage of replicate trees in which the associated taxa clustered together in the bootstrap test (1000 replicates) [33] is shown on each node. The evolutionary distances were computed using the Poisson correction method [34] and are presented in the unit of the number of amino acid substitutions per site. The analysis involved 32 amino acid sequences. There were 99 positions in each of the final datasets. Evolutionary analyses were conducted in MEGA11 [35]. Clusters with the six different MHC class I lineages found in teleosts are shown with colored boxes. The sequence references are listed in Figure 1.

Previous studies have described six MHC-I lineages in teleosts denoted by U, Z, S, L, P, and H. Using the Asian seabass α1 and α2 domain sequences (Figure 2), the sequences were used to draw a phylogenetic tree with various teleost fishes’ and human HLA. With bootstrap values ranging from 24–100%, the MHC-I lineage sequences form separate clusters. Phylogenetic tree analyses showed that the Asian seabass MHC-I sequences shared an overall specific similarity with the U sequences, forming a distinct cluster (Figure 3 and Figure S2).

### 2.3. Protein Modeling

**Figure 4:**
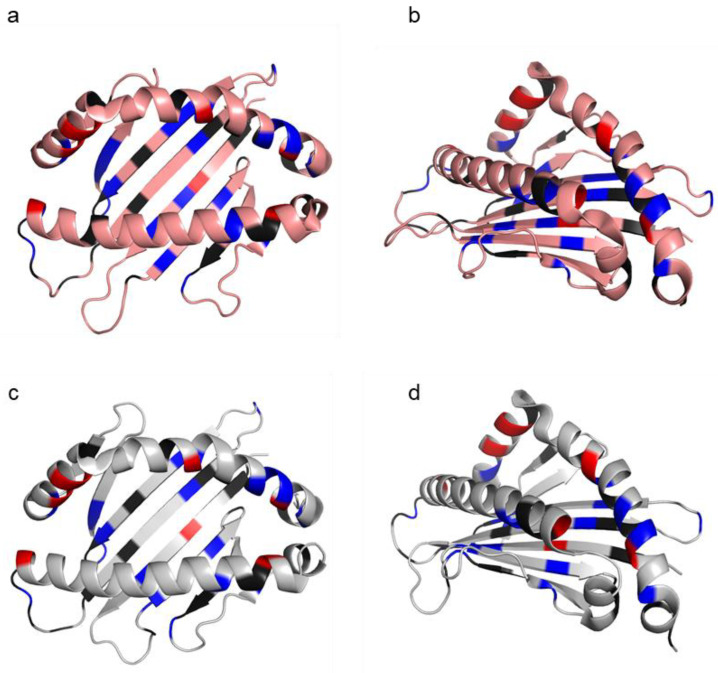
Schematic crystal structure of the human HLA-A*02:01 (PDB code 3MRE) and Ctid-UAA (PDB code 5H5Z) peptide binding groove defined by the α1 and α 2 domains. HLA-A*02:01 structure (**a**,**b**, pink) and Ctid-UAA structure (**c**,**d**, grey) with similar residues from the sequence of representative Asian seabass MHC-I residues mapped viewed from the top (**a**,**c**) and the PΩ side (**b**,**d**). Black indicates similar residues that were probably found before the molecular evolution of MHC classes I and II, blue indicates similar residues that are common in classical MHC-I, and red indicates conserved binding residues in classical MHC-I.

To date, the Asian seabass MHC-I crystal structure remains undefined. To understand how the Asian seabass binding residues map onto the classical MHC-I molecules, two high-resolution human and teleost fish crystal structures were selected for model building: HLA-A*02:01 structure (PDB code 3MRE) [36] and grass carp MHC-I (Ctid-UAA) structure (PDB code 5H5Z) [37]. The HLA-A2 family is the largest allele family of the HLA-A locus, with HLA-A*02:01 being the most predominant allele present in 50% of the population in the United States [38], while Ctid-UAA is the only teleost fish with available MHC-I structure [37]. The overlapping of the structures of HLA-A2 (pink) and Ctid-UAA (grey) shows that both molecules have similar structures (Figure S3). The conserved residues from the Asian seabass sequences were then highlighted on HLA-A2 and Ctid-UAA structures (Figure 4). The schematic indicates that the binding residues (red) of the Asian seabass MHC-I sequence were the same in both HLA-A2 and Ctid-UAA, with slight differences in the view from the top and the PΩ side of residues that were found before the molecular evolution of the MHC classes I and II (black) or that are common in classical MHC-I (blue) (Figure 4).

## 3. Discussion

MHC-I is an important immune molecule that is made up of highly polymorphic genes to enhance the recognition of pathogens aiding the resistance of hosts to infectious diseases. Often, MHC-I displays significant differentiation across geographical environments and populations, such as fishes [39,40,41,42,43,44]. Therefore, to characterize the MHC-I binding domains in Asian seabass, we randomly sampled 12 individual fishes. Comparing the Asian seabass MHC-I α1 and α2 domains, the sequence is more conserved where we identified 4 highly polymorphic sites with *V* > 1 in the α1 domain versus 16 polymorphic sites with *V* > 1 in the α2 domain. In contrast, sequence analyses of HLA-A revealed 6 highly polymorphic sites with *V* > 1 in the α1 domain and 7 polymorphic sites with *V* > 1 in the α2 domain from the 230 sequences obtained in the IMGT/HLA database [45,46].

Regardless of the genetic heterogeneity, the α1 and α2 domains consisted of eight evolutionary conserved residues that anchored the peptide [11,27]. The set of residues in mammals is YYYYYTKW, while that in non-mammalian vertebrates such as fishes is slightly different, YYYRFTKW [18,26]. Indeed, the sequence of the Asian seabass MHC-I contains all eight conserved binding residues, including R84. Further analysis of residues that were either present before the molecular evolution of MHC classes I and II or residues that were common to MHC-I revealed that the Asian seabass MHC-I possesses most of these residues. Moreover, by mapping these residues onto the HLA-A2 and Ctid-UAA crystal structures, we suggest that the Asian seabass MHC-I gene here is likely to function similarly to the classical MHC-I.

Phylogenetic analyses using neighbor-joining trees and maximum likelihood trees based on α1 and α2 domain amino acids suggest that the Asian seabass MHC-I gene sequences clustered close to the U lineages from different fish species. U lineage genes are predicted to provide the classical function of antigen presentation, which explains why the conserved residues were mostly found in these sequences. Interestingly, the Asian seabass MHC-I sequences consistently group with salmonid alleles and may indicate trans-species polymorphisms, which is common for MHC genes [47]. While these observations must be interpreted with caution as the bootstrapping support was relatively low, similar findings were reported in a recent study on the MHC-I alpha 1 domain in European seabass (*Dicentrarchus labrax*) [48].

In conclusion, we characterized a single MHC-I gene that belongs to the U lineage in Asian seabass. Currently, other than the MHC-I sequences that were obtained from an Asian seabass using next-generation sequencing, there is no known information regarding the polymorphism within its species and the genes have not been validated by another study. The availability of MHC-I α1 and α2 sequences enhances the quality of MHC class I genetic information in Asian seabass, provides new tools to analyze Asian seabass immunity against pathogenic infections, and will be applicable for phylogeny and evolution studies of antigen-specific receptors. Furthermore, the investigation of the MHC-I α1 and α2 sequences will help to uncover other polymorphisms at the interaction surfaces in Asian seabass. Future directions would include the search for experimental testing to generate three-dimensional structures and assess antigen binding interactions between MHC-I and T cell receptor molecules to modulate Asian seabass immune responses.

## 4. Materials and Methods

### 4.1. Materials

The genomes used in this study are as follows: Sasa-ZAAa *Salmo salar* (Atlantic salmon; accession ACX35596.1) [49], Leoc-LO14 *Lepisosteus oculatus* (spotted gar; accession GFIM01040660) [21,50], Sasa-SAA Salmo *salar* (Atlantic salmon; accession ACY30362.1) [49], Asme-AM33 *Astyanax mexicanus* (Mexican tetra; accession KB882192.1) [21], Sasa-LCA *Salmo salar* (Atlantic salmon, accession XP_013983104) [21,51], *Leoc-LO12 Lepisosteus oculatus* (spotted gar; accession JH591577) [21], Taru-TR6 *Takifugu rubripes* (fugu; accession HE591930) [21,52], Asme-P *Astyanax mexicanus* (Mexican tetra; accession GFIF01000014) [50], Sasa-UBA *Salmo salar* (Atlantic salmon; accession XP_014032819.1) [51], Leoc-U *Lepisosteus oculatus* (spotted gar; accession GFIM01032149) [50], Ctid (*Ctenopharyngodon idella*, Grass carp) -UAA (5H5Z-A) [37], HLA-A2:01 *Homo sapiens* (human; accession AAA76608.2) [53], HLA-B15:01 *Homo sapiens* (human; accession HG794370.1), HLA-C1:02 *Homo sapiens* (human; accession HG794388.1), and the predicted *Lates calcarifer* class I histocompatibility antigen, F10 alpha chain-like (Asian seabass; accession XM_018684417) [22].

### 4.2. Data Mining

*Lates calcarifer* MHC-I gene sequences were identified using known bony fish and human MHC gene sequences [21,49,50,51,52] and tblastn against annotated databases available in the Ensembl and NCBI databases.

### 4.3. Fish Samples

Farmed Asian seabass, SG1-SG9 and AU1-AU3, were collected from a commercial Singapore farm and MainStream Australia, respectively. The fishes sampled were between 40 and 119 days old, with weights ranging from ~2 g to 61 g and lengths ranging from ~2 cm to 15 cm.

### 4.4. Experimental Analyses of Asian Seabass Mhc-I α1 and α2 Domains Sequence

Total RNA was isolated from Lates calcarifer (Asian seabass) muscle tissues using an Rneasy mini kit (Qiagen, Hilden, Germany) and transcribed into cDNA using oligo (dT) with a SuperScript III First-Strand Synthesis system (Invitrogen, Waltham, MA, USA) as per the manufacturer’s instructions. The coding sequence of the Asian seabass MHC-1 α1 and α2 domain genes was amplified by Phusion Hot Start II High-Fidelity DNA Polymerase (Thermo Scientific, Waltham, MA, USA) using a forward primer, LCA-MHC-1-F1 (5′-TTCCTGTTCCTGTGCCACG-3′), and a reverse primer, LCA-MHC-1-R1 (5′-AGAGACACTGAGGGAAGGGC-3′), designed at exon 1 and exon 4, respectively, of the RefSeq Genome sequencing and assembly PRJNA345597. The PCR reaction contained 10 µL of 5X Phusion HF buffer, 200 µM of dNTPs, 0.5 µM of each primer, 0.5 µL of Phusion Hot Start II DNA polymerase, and 5 µL of cDNA in a total volume of 50 µL. The PCR conditions were denaturation at 98 °C for 1 min, 35 cycles of denaturation at 98 °C for 15 s, annealing at 60 °C for 30 s, elongation at 72 °C for 30 s, and final elongation at 72 °C for 7 min. The PCR product size was determined using gel electrophoresis (~700 bp; Figure S4), and the PCR products were purified with a Monarch^®^ PCR & DNA Cleanup kit (New England Biolabs, Ipswich, MA, USA). DNA sequencing was performed by Bio Basic Asia Pacific Pte Ltd. (Singapore) using the forward primer LCA-MHC-1-F1 (5′-TTCCTGTTCCTGTGCCACG-3′) and reverse primer LCA-MHC-1-R2 (5′-TCCCAGTACTCAGGTCCTTC-3′). The 12 sequences obtained in this study were deposited at GenBank as accessions OP348933 to OP348944, respectively.

### 4.5. Sequence Alignments, Variability, and Phylogenetic Analysis

MHC-I amino acid sequences were aligned using Clustal Omega [54] for initial analyses and manually curated subsequently in Jalview [55] for some predicted sequences. Pairwise alignment was conducted using Jalview [55] and plotted as heatmaps with the mean ± s.e. calculated using Graphpad Prism 9. The MHC-I α1 and α2 sequence variability and polymorphisms were determined using variability metric (*V*) analysis [46]. The variability metric is based on the Shannon entropy equation and calculated as $V=-\overset{M}{\underset{t=1}{\sum }}P_{i}{log}_{2}P_{i}$, where P_i_ is the fraction of residues of amino acid type *i* and *M* is the number of amino acid types [46]. *V* ranges from 0 (most conserved site with only one amino acid type at that position) to 3.7 (most variable site with all 13 amino acids represented differently at that position). Sites with *V* > 1 are considered to be highly polymorphic. For phylogenetic tree analysis, the α1 and α2 domains were inferred using the neighbor-joining method [32], with bootstrap testing according to Felsenstein [33]. The evolutionary distances were computed using the Poisson correction method and were presented in the unit of the number of amino acid substitutions per site [34]. All ambiguous positions were removed for each sequence pair. There were 99 positions in each of the final datasets. The lineage clustering was further supported by another phylogenetic tree constructed using the maximum likelihood method based on the optimal JTT matrix-based model [56]. Evolutionary analyses were conducted in MEGA11 [35].

### 4.6. Three-Dimensional Modeling of Human HLA-A2-01 and Grass Carp MHC-I α1 and α2 Domains

The structures of HLA-A*02:01 (PDB code 3MRE) [36] and Ctid-UAA (PDB code 5H5Z) [37] were studied and contrasted using The PyMol Molecular Graphics System, Version 2.1.1 Schrödinger, LLC, New York, NY, USA.

## Data Availability

Data are contained within the article or Supplementary Material.

## Supplementary Materials

The supporting information can be downloaded at: https://www.mdpi.com/article/10.3390/ijms231810688/s1. References [35,56] are cited in the supplementary materials.
