# Supplementary material for: Molecular Characterization of MHC Class I Alpha 1 and 2 Domains in Asian Seabass (Lates calcarifer)
Source: Int J Mol Sci. 2022 Sep 14;23(18):10688. doi: 10.3390/ijms231810688 (PMC9500968; doi:10.3390/ijms231810688)
Supplement: Supplementary file 1 [file ijms-23-10688-s001.zip › ijms-1831385-supplementary.pdf]

Supplementary Materials

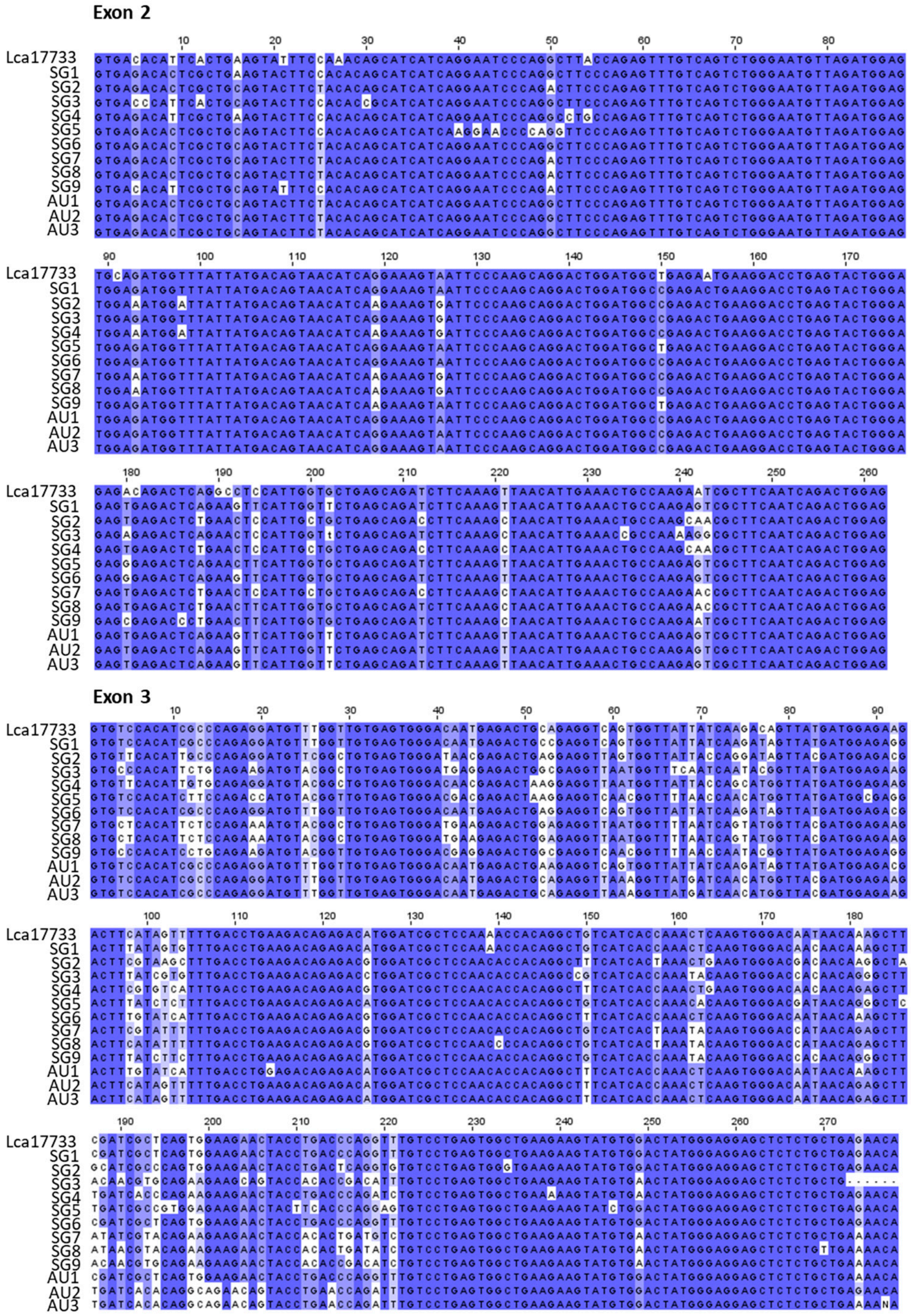

Figure S1. Asian seabass Mhc-I exon 2 and exon 3 DNA analysis and sequencing. Multiple sequence alignments of 12 Asian seabass Mhc-1 exon 2 and exon 3 domains sequences against Lca17733 sequence obtained from RefSeq Genome sequencing and assembly PRJNA345597. The numbering corresponds to the position within Lca17733. Identity with consensus is shown in blue. SG denotes samples obtained from Singapore and AU denotes samples obtained from Australia.

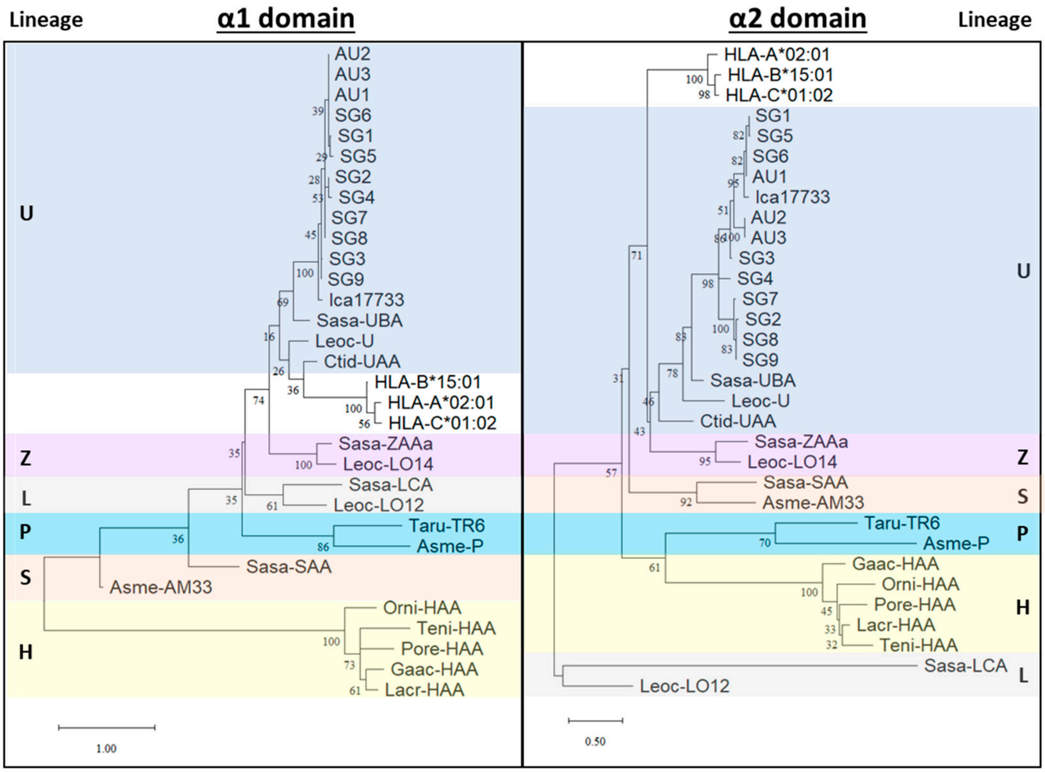

Figure S2. Phylogenetic trees based on $\alpha 1$ and $\alpha 2$ domain amino acid sequences of Asian seabass and other representative teleost fishes MHC-I molecules. A Maximum Likelihood tree of $\alpha 1$ (left) and $\alpha 2$ (right) domain sequences. The tree with the highest log likelihood [-2155.05 (left) and -3411.87 (right)] is shown. The percentage of trees in which the associated taxa clustered together is shown below the branches. Initial tree(s) for the heuristic search were obtained automatically by applying Neighbor-Join and BioNJ algorithms to a matrix of pairwise distances estimated using the JTT model [56], and then selecting the topology with superior log likelihood value. A discrete Gamma distribution was used to model evolutionary rate differences among sites [5 categories (+G, parameter = 7.7889 (left tree) and 6.0639 (right tree)]. The rate variation model allowed for some sites to be evolutionarily invariable ([+I], 1.75% sites (left tree) or 1.20% sites (right tree)). The tree is drawn to scale, with branch lengths measured in the number of substitutions per site. This analysis involved 32 amino acid sequences. All positions with less than 95% site coverage were eliminated if fewer than 5% alignment gaps, missing data, and ambiguous bases were allowed at any position. There was a total of 57 positions (left tree) and 83 positions (right tree) in the final dataset. Evolutionary analyses were conducted in MEGA11 [35].

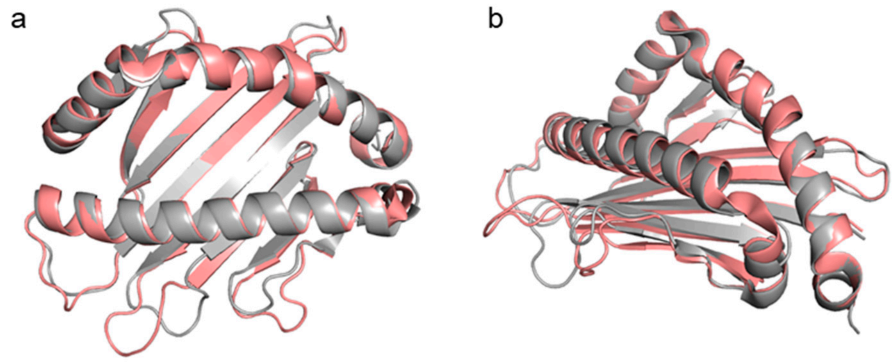

Figure S3. Schematic crystal structure of overlapped human HLA-A*02:01 (PDB code 3MRE) and Ctid-UAA (PDB code 5H5Z) peptide binding groove defined by the α1 and α 2 domains. The main chains of HLA-A*02:01 (pink) and Ctid-UAA structure (grey) depicted schematically as cartoons were mapped onto viewed from the top (**a**) via the PΩ side (**b**).

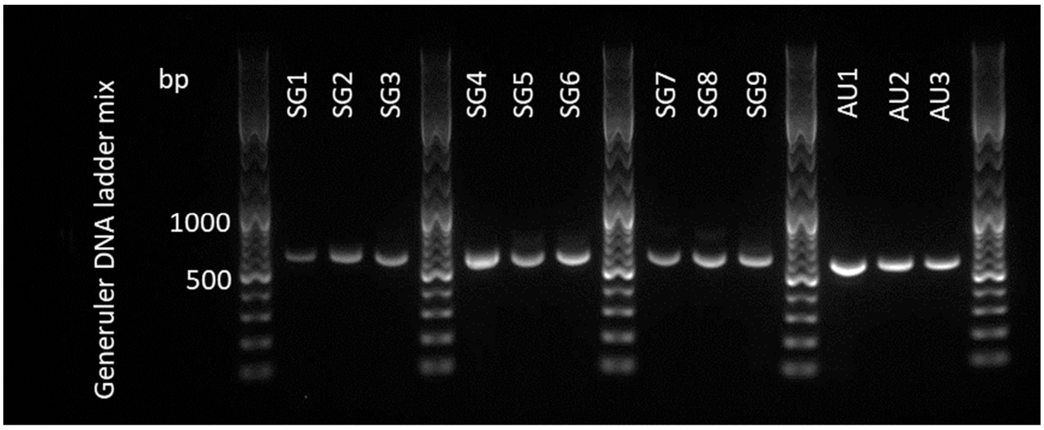

Figure S4. Asian seabass Mhc-I exon 2 and exon 3 PCR products. (a) PCR products from Asian seabass muscle tissues were amplified with Lca-Mhc-1-F1 and Lca-Mhc-1-R1 primers on a 1% agarose gel. DNA was used at 1/10 dilution in the PCR reaction. DNA ladder mix (Generuler) was used to determine band sizes given in base pairs. A product of approximately 700 bp was amplified from all samples. SG denotes samples obtained from Singapore and AU denotes samples obtained from Australia.
